# A Simple Predictive Factor for Mortality in Fontan Surgery: Serum Hypo-Osmolality

**DOI:** 10.21470/1678-9741-2019-0325

**Published:** 2020

**Authors:** Baburhan Ozbek, Nursen Tanrıkulu

**Affiliations:** 1 Department of Pediatric Cardiovascular Surgery, Van Training and Research Hospital, Van, Turkey.; 2 Department of Anesthesiology, Kolan International Hospital, Istanbul, Turkey.

**Keywords:** Fontan Procedure, Mortality, Postoperative Period, Exudates and Transudates, Osmolar Concentration, Pleural Effusion, Cardiac Output

## Abstract

**Objective:**

Close follow-up is important after the Fontan procedure, which is a palliative surgical method for a single ventricle. In this period, serum osmolality is an important parameter with the advantages of easy to obtain and poor outcome prediction.

**Methods:**

Patients who had undergone Fontan operation between May 2011 and February 2017 were retrospectively evaluated. Patients were divided into three groups based on their serum osmolality values: hypoosmolar (Group 1), isosmolar (Group 2), and hyperosmolar (Group 3). Demographics, clinical information and postoperative data of the groups were compared.

**Results:**

Forty-three patients had undergone extracardiac Fontan operation in the study period. There were 8, 19 and 16 patients in Groups 1, 2 and 3, respectively. Among the three groups, postoperative intubation and length of hospital stay, prolonged pleural effusion, need for inotropic support and mortality were statistically significantly higher in Group 1.

**Conclusion:**

After the Fontan procedure, one of the determinants of cardiac output might be affected by serum osmolality. Decreased serum osmolality might be associated with poor prognosis after Fontan procedure. Serum osmolality monitoring may be beneficial to improve postoperative outcomes in these patients.

**Table:** 

Abbreviations, acronyms & symbols	
CPB	= Cardiopulmonary bypass
BUN	= Blood urea nitrogen
ECMO	= Extracorporeal membrane oxygenation
PAP	= Pulmonary artery pressure

## INTRODUCTION

Functional single ventricle constitutes 10% of all congenital heart disease and Fontan procedure became the milestone in palliative surgical treatments of patients with a single ventricle after 1971^[1-3]^. The main objective of the Fontan procedure is to form balance by distinguishing systemic and pulmonary circulation with cavopulmonary connection^[4]^.

Patients with a single functional ventricle may have different clinical symptoms such as cyanosis (a sign of pulmonary circulation problem) or nutrition (a sign of systemic circulation problem) problems, hypotension or postpartum shock^[5]^. Functional left ventricle can be diagnosed with fetal echocardiography, magnetic resonance imaging or computed tomography. Magnetic resonance plays an important role, especially in screening of pulmonary vascular structures and in evaluating the adequacy of ventricular and arterioventricular valves^[6,7]^. Cardiac catheterization is used to measure pressures and screening of pulmonary artery, aortic arch and their branches. Close medical follow-up is required for these patients before and after the surgical procedure.

Serum osmolality, which can be defined as the soluble load in the body, is one of the easy and important parameters in the follow-up period. There are studies reporting a close relationship between serum osmolality and inflammatory process^[8]^. Cardiac output is related to the flow rate of Fontan circulation after the Fontan procedure. Volume replacement is required to increase cardiac output in the patient with Fontan procedure, which works worse than expected. This concludes the decrease in serum osmolality. In the evaluation of more than 4,000 patients, hyponatremia, determinant of serum osmolality, both preoperatively and postoperatively, was found as a predictive factor for mortality^[9,10]^. In this study, we evaluated the effects of serum osmolality in patients with a single ventricle who had undergone the Fontan procedure.

## METHODS

Patients who had undergone Fontan surgery between May 2011 and February 2017 in our department were retrospectively analyzed. Demographic records, clinical and laboratory findings were obtained from medical records. We included all patients in this period and divided them into 3 groups according to preoperative serum osmolality. Patients with <280 serum osmolality (hypoosmolality) were in Group 1, patients with serum osmolality values between 280 and 295 (isosmolality) were in Group 2, and patients with serum osmolality values ˃295 (hyperosmolality) were in Group 3.

Before the operation, pressure measurements were evaluated in all patients with catheterization. In all patients, extracardiac Fontan operation was performed under standard cardiopulmonary bypass (CPB) procedure. Through median sternotomy, ascending aorta, superior and inferior vena cavae, pulmonary arteries and, if previously applied, pulmonary shunts of the aorta and Glenn shunts were released. Pulmonary artery pressure was measured and checked invasively, and the operation was continued in patients with PAP <18 mmHg. Antegrade pulmonary flow was prevented if present. Ascending aorta was preferred for arterial cannulation and innominate vein and inferior vena cava were preferred for venous cannulation. Cardiac arrest was induced with antegrade blood cardioplegia in cases requiring aortic cross clamping such as atrioventricular valve intervention or atrial septectomy. During the extracardiac Fontan procedure, tube graft was anastomosed to the inferior vena cava and pulmonary artery with continuous suture technique, respectively. While separating the vena cava from right atrium, attention was paid to avoid damage to coronary sinus or coronary artery. Four-millimeter punch was used for fenestration between the graft and the right atrium.

In patient follow-up, we try to maintain balance with adequate diuresis and <3 lactate levels. To provide cardiac output, we especially use colloidal fluids. We performed extracorporeal membrane oxygenation (ECMO) in the persistence of low cardiac output syndrome, despite volume replacement and inotropic support.

Statistical analyses were performed with IBM Statistical Package for the Social Sciences 24.0 software (SPSS 24.0, SPSS Inc., Chicago, IL). Categorical variables are summarized as number (percentage) and continuous variables are summarized as mean ± standard deviation, as appropriate. Groups were compared using Student’s t-test and Wilcoxon test.

## RESULTS

Forty-three patients had undergone extra-cardiac Fontan operation from May 2011 to February 2017 in our department. Among these patients, 8 were in Group 1 (19%), 19 in Group 2 (44%) and 16 patients in Group 3 (37%). Forty-two percent of patients were male (n=18) and 58% (n=25) were female, aged from 22 months to 23 years. Preoperative variables are detailed in Table 1.

**Table 1:** Comparison of demographic data and preoperative variables.

Variable	Group 1 (n=8)	Group 2 (n=19)	Group 3 (n=16)	*P*-value
Median age (years)	8.2±1.4	7.5±2.3	8.9±1.2	0.915
Gender (male/female)	03-May	08-Nov	07-Sep	1.000
Average weight (kg)	23.5±4.4	22.4±5.1	26.7±3.5	0.651
Left ventricular dominancy (n)	5 (62.5%)	12 (63.1%)	11 (68%)	0.213
Mean PAP (mmHg)	13.5±1.9	13.2±2.3	12.8±2.5	0.790
Oxygen saturation (%)	77.9±3.1	78.3±2.5	80.1±2.2	0.529
Serum albumin (g/dl)	3.5±0.9	3.6±0.5	3.7±0.4	0.933
Serum glucose (mg/dl)	90.2±35	101.5±28	114.4±21.2	0.147
Serum creatinine (mg/dl)	0.6±0.28	0.7±0.25	0.8±0.19	0.615
Serum urea (mg/dl)	27±4.9	28±3.7	29.4±3.2	1.000
Serum sodium (mg/dl)	133±3.7	138±2.9	142±2.1	0.038
Hemoglobin (gr/dl)	12.6±2.2	13.4±1.5	13.5±1.3	0.740
INR	1.1±0.4	1.21±0.22	1.2±0.3	0.809
Platelet level (×10^3^)	121±24	130±17	140±12	0.907
CRP	3.25±0.7	2.94±0.8	2.73±1.1	0.140

CRP=C reactive protein; INR=international normalized ratio; PAP=pulmonary arterial pressure

Preoperatively, the most common diagnosis was tricuspid atresia (60%). Aortopulmonary shunt was performed before Glenn procedure in 23 patients (53.5%). Four patients had no history of operation before Fontan procedure (9.3%). We applied pulmonary reconstruction in six patients (14%), common atrioventricular valve repair (14%) in 9 patients and atrial septectomy in 4 patients (9.3%) concomitantly with Fontan procedure. Extracardiac Fontan operation was performed with bidirectional cavopulmonary anastomosis in 5 patients (11.6%). The size of the tube grafts was 16 mm in 1 patient (2.3%), 18 mm in 19 patients (44%), 20 mm in 13 patients (30.2%), 22 mm in 7 patients (16.2%), and 24 mm and 3 patients (6.9%). Fenestration was applied during the operation in 9 patients (20.9%). However, postoperative fenestration was required in two patients who did not present fenestration during the operation in Group 1. In the comparison of operative data, there was no statistically significant difference among the groups (Table 2). Inotropic agent usage during surgery was statistically significantly higher in Group 1 (P=0.018).

**Table 2:** Comparison of operative data.

Variable	Group 1 (n=8)	Group 2 (n=19)	Group 3 (n=16)	*P*-value
ACC time (minutes)	45.6±14.3	44.3±16.3	42.3±17.2	0.725
CPB time (minutes)	92.5±15.4	88.5±18.3	87.5±22.6	0.170
Hypothermia temperature (ºC)	32.3±2.0	33.2±1.3	34.1±1.0	1.000
Preoperative PAP (mmHg)	14.3±1.6	13.5±1.9	12.8±2.3	0.253
Mean intraoperative Hb (mg/dl)	8.9±1.8	9.5±1.5	9.7±1.2	0.430
Amount of blood products used (ml)	250±90	260±80	280±40	1.000
Mean intraoperative glucose (mg/dl)	140±41	154±36	162±25	0.807
Fenestration procedure (n)	2 (25%)	4 (21.05%)	3 (19%)	0.625
Need for inotropic support (%)	5 (62.5%)	7 (36.8%)	5 (31.2%)	0.018

ACC=aortic cross clamp; CPB=cardiopulmonary bypass; HB=hemoglobin; PAP=pulmonary arterial pressure

In the follow-up period, supraventricular tachycardia attacks were revealed in 6 patients and all returned to sinus rhythm with medical treatment. There was no reoperation due to bleeding and no enteropathy causing protein loss in the short-term period. Four patients (9.3%) required ECMO support, 1 (12.5%) in Group 1, 2 (10.5%) in Group 2 and 1 (6.5%) in Group 3. All ECMO-supported patients died. Mortality was present in 6 patients (14%). The cause of mortality was low cardiac output in 3 patients, sepsis in 2 patients and graft thrombosis in 1 patient.

Postoperative general balance in Group 1 shifted to positive in order to increase cardiac output. Need for colloidal fluid was highest in Group 1. A statistically significant difference was detected among all groups on 1^st^ postoperative day regarding general balance and sodium levels. In the comparison of postoperative data, intubation and hospitalization times, prolonged pleural effusion rate (˃4 days) and mortality were statistically significantly higher in Group 1. There was a statistically significant difference in serum sodium and hemoglobin levels in Group 1 because of volume replacement, but there was no significant difference for blood replacement (Table 3).

**Table 3:** Comparison of postoperative data.

Variable	Group 1 (n=8)	Group 2 (n=19)	Group 3 (n=16)	*P*-value
Intubation time (hours)	11.7±5.2	8.6±3.4	7.8±4.1	0.016
Drainage (ml)	350±40	380±30	290±70	0.390
SVT (n)	1 (12.5%)	3 (15.7%)	2 (12.5%)	0.170
Mean glucose on 1^st^ PO day (mg/dl)	137±44	145±32	154±27	1.000
Balance on 1^st^ PO day (ml)	390±70	180±30	110±50	0.0003
Serum Na on 1^st^ PO day	132±4.1	137±3.2	143±2.9	0.026
Postoperative hemoglobin (mg/dl)	9.6±1.5	10.8±1.7	11.4±1.2	0.028
CRP (mg/l)	92.27±19	85.63±24	79.41±26.2	0.095
Amount of blood products used (ml)	260±30	280±20	230±50	0.903
Drain removal (day)	4.3±0.9	3.4±0.5	3.2±0.7	0.034
Prolonged pleural effusion (n)	4 (50%)	4 (21%)	3 (18%)	0.009
Length of stay in intensive care (days)	5.8±1.4	4.2±1.1	3.7±1.3	0.026
Hospitalization time (days)	12.2±1.9	10.2±1.3	9.4±1.6	0.042
ECMO requirement (n)	1 (12.5%)	2 (10.5%)	1 (6.5%)	0.075
Mortality (n)	3 (37.5%)	2 (10.5%)	1 (6.25%)	0.014

CRP=C reactive protein; ECMO=extracorporeal membrane oxygenation; PO=postoperative; SVT=supraventricular tachycardia

## DISCUSSION

Serum osmolality, defined as the solute load in the body, can be calculated with the 2 × Na + Glucose/18 + Blood urea nitrogen/2.8 formula. Its normal value is 280-295 mOsm/kg H2O^[11]^. There are studies reporting a close relationship between serum osmolality and inflammatory process^[8,12]^. Fluid and electrolyte disturbance might cause severe cardiac rhythm disorders and altered cardiac loading^[13]^. In the evaluation of 315 patients, Rasouli et al.^[14]^ reported a connection between serum osmolality and coronary artery disease. In this study, we investigated the effect of serum osmolality on extracardiac Fontan operations by comparing 3 groups. To our knowledge, there is no similar study in the literature that looks at the effect of serum osmolality in patients with a functional singular ventricle. In our study, requirement of inotropic support, postoperative intubation and hospitalization period, prolonged pleural effusion and mortality were higher in patients with low serum osmolality. Although serum C reactive protein levels were higher in Group 1, it did not reach a statistically significant level. Correlation between serum osmolality and inflammation might be found in further studies with larger series.

Total cavopulmonary connection might be performed as intracardiac, extracardiac or intra and extracardiac technique. Lateral tunnel technique, which is an intracardiac approach, and extracardiac Fontan technique are mostly preferred procedures^[15]^. These two techniques have superiorities among themselves. The growth potential is not limited in the lateral tunnel technique and fenestration can be done faster. In extracardiac Fontan technique, there is no intracardiac suture line or prosthetic material that might increase the risk of arrhythmia and there is no need for aortic cross clamping. Energy loss is decreased by providing laminar flow and lower venous pressure values are provided^[16]^. We applied extracardiac Fontan procedure in our cases. We prefer the largest tubular graft as we might use considering the body size in the operation.

Potential side effects of CPB are increased pleural effusion and prolonged mechanical ventilatory support^[17]^. In contrast, there are also articles reporting that CPB has no effect on morbidity and mortality with extracardiac Fontan operation^[18]^. In the evaluation of 285 patients, Petrossian et al.^[19]^ declared that the usage of CPB does not affect early and mid-term results of extracardiac Fontan procedure. We performed our operations under the guidance of CPB. However, we avoid deep hypothermia due to its potential side effects such as increased pleural effusion and longer intensive care and hospital stay.

In Fontan procedure, aortic cross clamping, right ventricular dominance, heterotaxia syndrome, common atrioventricular valve intervention, unprovided sinus rhythm, postoperative left atrial pressure >13 mmHg and pulmonary artery pressure >20 mmHg were reported as poor prognostic factors^[20]^. There was no statistically significant difference about left ventricular dominance, pulmonary artery pressure and prevalence of supraventricular tachycardia among the groups in our study. Pulmonary artery pressure was below 18 mmHg in all patients to protect the homogeneity of groups.

During the Fontan procedure, application of fenestration has a positive effect on patients with pulmonary artery pressure of 15-18 mmHg^[21]^. Although low saturation is observed after fenestration, it provides increased cardiac output and decreased central venous pressure. We performed fenestration in 9 patients (20.9%) with high pulmonary artery pressure. There was no statistically significant difference among the groups in patients who had operative fenestration. However, in Group 1, 2 cases required fenestration after the operation.

Serum sodium plays an important role in the regulation of cellular functions and might have effects on the functions of cardiomyocytes. Preoperative hyponatremia is associated with longer hospital stays, higher readmission rate, and shorter longterm survival^[9]^. Preoperative serum sodium levels were normal in our patients. Postoperative diuretic treatment, general balance, antidiuretic hormone activity, new onset of congestive heart failure or renal failure, might be the cause of hyponatremia^[10]^. Hyponatremia rate in Fontan patients was reported to be 30% due to high dose of diuretics, elevated renin activity and norepinephrine levels, and was reported as an independent predictor of unscheduled readmissions. Activation of the reninangiotensin-aldosteron system with negative sodium balance by the use of diuretics is the main cause of hyponatremia in pediatric patients^[22]^.

In Fontan physiology, pulse rate, which is one of the two components of cardiac flow, is losing its importance and pulse volume becomes prominent. The most important indicator of pulse volume as osmolality is sodium. Therefore, an optimal sodium level is required to provide cardiac output and flow. In this way, intravascular volume and cardiac output are better protected. Hyponatremia will increase the body volume to protect the pulse volume that causes edema. It is difficult to maintain cardiac flow in hyponatremia and the increased volume causes systemic venous hypertension. As a result, Fontan circulation cannot work efficiently, resulting in increased mortality. In our study, we revealed a correlation between serum hypoosmolality and mortality, which is similar to the serum sodium levels on the 1^st^ postoperative day. Perioperative evaluation and treatment of hypoosmolality might improve postoperative outcomes after the Fontan procedure.

## CONCLUSION

Serum osmolality, a simple and cost-effective clinical variable, can predict mortality risk during the Fontan procedure. With the increased awareness of serum osmolality on mortality, preoperative causes of serum hypoosmolality might be investigated, and some might be avoided to decrease postoperative mortality. Multicenter prospective studies, including a larger number of patients, are needed to determine serum hypoosmolality and its effect on the postoperative outcomes of patients with Fontan procedure.

**Table:** 

Authors' roles & responsibilities	
BO	Substantial contributions to the conception or design of the work; or the acquisition, analysis or interpretation of data for the work; drafting the work or revising it critically for important intellectual content; agreement to be accountable for all aspects of the work in ensuring that issues related to the accuracy or integrity of any part of the work are appropriately investigated and resolved; final approval of the version to be published
NT	Drafting the work or revising it critically for important intellectual content; agreement to be accountable for all aspects of the work in ensuring that issues related to the accuracy or integrity of any part of the work are appropriately investigated and resolved; final approval of the version to be published
